# Development of a universal medical X‐ray imaging phantom prototype

**DOI:** 10.1120/jacmp.v17i6.6356

**Published:** 2016-11-08

**Authors:** Annemari Groenewald, Willem A. Groenewald

**Affiliations:** ^1^ Equra Health Vergelegen Oncology Unit 1 Arun Place, Sir Lowry's Pass Road Somerset West South Africa; ^2^ Department of Medical Imaging and Clinical Oncology Stellenbosch University and Tygerberg Academic Hospital Cape Town South Africa

**Keywords:** image quality assurance, phantom, diagnostic radiology, X‐ray imaging, prototype

## Abstract

Diagnostic X‐ray imaging depends on the maintenance of image quality that allows for proper diagnosis of medical conditions. Maintenance of image quality requires quality assurance programs on the various X‐ray modalities, which consist of projection radiography (including mobile X‐ray units), fluoroscopy, mammography, and computed tomography (CT) scanning. Currently a variety of modality‐specific phantoms are used to perform quality assurance (QA) tests. These phantoms are not only expensive, but suitably trained personnel are needed to successfully use them and interpret the results. The question arose as to whether a single universal phantom could be designed and applied to all of the X‐ray imaging modalities. A universal phantom would reduce initial procurement cost, possibly reduce the time spent on QA procedures and simplify training of staff on the single device. The aim of the study was to design and manufacture a prototype of a universal phantom, suitable for image quality assurance in general X‐rays, fluoroscopy, mammography, and CT scanning. The universal phantom should be easy to use and would enable automatic data analysis, pass/fail reporting, and corrective action recommendation. In addition, a universal phantom would especially be of value in low‐income countries where finances and human resources are limited. The design process included a thorough investigation of commercially available phantoms. Image quality parameters necessary for image quality assurance in the different X‐ray imaging modalities were determined. Based on information obtained from the above‐mentioned investigations, a prototype of a universal phantom was developed, keeping ease of use and reduced cost in mind. A variety of possible phantom housing and insert materials were investigated, considering physical properties, machinability, and cost. A three‐dimensional computer model of the first phantom prototype was used to manufacture the prototype housing and inserts. Some of the inserts were 3D‐printed, others were machined from different materials. The different components were assembled to form the first prototype of the universal X‐ray imaging phantom. The resulting prototype of the universal phantom conformed to the aims of a single phantom for multiple imaging modalities, which would be easy to use and manufacture at a reduced cost. A PCT International Patent Application No. PCT/IB2016/051165 has been filed for this technology.

PACS number(s): 87.57.C, 87.59.‐e

## I. INTRODUCTION

Different imaging modalities are used in diagnostic radiology to diagnose and follow up a variety of disease conditions. In order to ensure that the images are of acceptable quality for accurate clinical diagnosis, image quality has to be evaluated and maintained. Image quality is a subjective concept that requires certain measures in order to be quantified; by using a phantom, for example. Currently modality‐specific phantoms are used for image quality assurance in each of these imaging modalities.

The commercially available phantoms for mammography include the American College of Radiology (ACR) mammography phantom that contains fibres (1.56, 1.12, 0.89, 0.75, 0.54, and 0.40 mm in diameter) and simulates tumorous masses with 2.00, 1.00, 0.75, 0.50, and 0.25 mm diameter hemispheres, as well as micro‐calcifications with 0.54, 0.40, 0.32, 0.24, and 0.16 mm speck groups.^(1)^ The ACR mammography phantom is 4.2 cm thick and consists of 3.5 cm Lucite and a 0.7 cm thick paraffin insert, which contains the image quality indicators.^(2)^ The NORMI PAS phantom (PTW‐Freiburg GmbH, Freiburg, Germany) is used for image quality assurance on digital mammography units. The base plate of the phantom is semicircular to simulate breast shape. Two rows of balls are used at chest‐wall side to investigate image alignment. An aluminium step‐wedge can be placed in a cutout in the base plate. The step‐wedge consists of 14 steps 0–5.2 mm in thickness for sensitometry evaluation. Alternatively, a polymethyl methacrylate (PMMA) step‐wedge with 14 steps of 0–39 mm thickness can be inserted in the cutout. Different test elements can be fitted into the cutout in the structure layer. The PMMA test element is used to assess optical density in a region of interest (ROI). The signal‐difference‐to‐noise ratio (SDNR) is calculated from the SDNR test element, which is used to measure average pixel values for the calculation. The ACR test element contains fibres, microcalcifications, and tumorous masses for visual image quality evaluation. Fibres have diameters of 1.5, 1.1, 0.9, 0.7, 0.55, and 0.4 mm. Masses have thicknesses of 2.0, 1.0, 0.75, 0.5, and 0.25 mm. The speck groups used to simulate microcalcifications are 0.5, 0.4, 0.3, 0.2, and 0.12 mm in diameter. A dose detector can also be fitted in the phantom.^(3)^

For CT scanning the Catphan CT phantom (The Phantom Laboratory, Greenwich, NY) has two low‐contrast modules. The supra‐slice region has three groups of inserts, each with nine circular objects with diameters between 2 and 15 mm and contrast of 0.3%, 0.5%, and 1.0%. In the subslice module three groups of four inserts each are contained. Diameters range between 3 and 9 mm and contrast is fixed at 1.0%.^(4)^ The Gammex ACR CT phantom (Gammex RMI, Middleton, WI) is made from Solid Water and has a 20 cm diameter and 16 cm length. It contains water‐equivalent, bone‐equivalent, acrylic, air, and polyethylene inserts for CT‐number linearity assessment. A 0.011 mm diameter tungsten carbide bead is used for modulation transfer function (MTF) calculation. Aluminium and polystyrene line pair material is used for resolution assessment with bar phantoms 4, 5, 6, 7, 8, 9, 10, and 12 line pairs per centimetre (lp/cm). Steel balls of 1 mm diameter are used for positioning and alignment checks and 0.28 mm ball bearings for distance measurements on an axial slice. A low‐contrast rod module is used for low‐contrast resolution with 6, 5, 4, 3, and 2 mm diameter cylinders at a contrast 0.6% different from background. Four cylinders of each diameter are included. CT‐number uniformity is assessed with ROI analysis. Slice thickness is checked with two wire ramps evident in 0.5 mm z‐axis increments.^(5)^

The CIRS Model 903 phantom (Radcal Corporation, Monrovia, CA) is used in radiography. It has low‐contrast evaluation holes in an aluminium disk at 9.5 mm diameter and 1.73, 1.24, 0.89, 0.64, 0.46, 0.32, 0.23, 0.16, and 0.10 mm depths. High contrast is assessed with a mesh of 0.47, 0.63, 0.79, 0.94, 1.18, 1.57, 1.97, 2.36, and 3.15 line pairs per millimetre (lp/mm) and also contains a contrast detail insert.^(6)^ The NORMI 13 phantom (PTW‐Freiburg GmbH) is designed for acceptance and constancy tests for digital projection radiography. The phantom tests signal standardization by measuring the brightness of an image at a central area. It has seven dynamic steps, consisting of different thickness copper plates from 0.0 mm to 2.3 mm, for evaluation of contrast resolution. The steps should be separately identifiable. For low‐contrast evaluation, six disks with contrasts of 0.8% to 5.6% are visually inspected at an image window setting where all seven dynamic steps are depicted differently. For homogeneity the optical density or luminance is measured in five different areas, at the center and at the four corners of the image. A lead foil test pattern is used to evaluate spatial resolution, using a magnifying glass. Image geometry is assessed by measuring the distances between different lines. These lines are also used to assess scaling and are checked for distortions. The variation between the light field and X‐ray field is investigated using the different field size radiopaque field edge marker lines. The image is also evaluated for the presence of artifacts. An additional copper plate is supplied for use with higher kV setting (e.g., 100 kV). The phantom can also evaluate delivered radiation dose by establishing a dose indicator versus image brightness relationship at acceptance testing.^(7)^

The TOR CDRH Fluoroscopic phantom (Leeds Test Objects, Broughbridge, UK) has eight low‐contrast test holes of the same diameter, 9.5 mm, and depths ranging between 0.16 to 1.73 mm.^(8)^ The NORMI Rad/Flu phantom (PTW‐Freiburg GmbH) is used for acceptance and constancy testing in fluoroscopy. It incorporates a copper step‐wedge for sensitometry assessment, a resolution test pattern, a grid plate, and eight low‐contrast detection inserts.^(9)^ The resolution is assessed visually by reading the lp/mm resolved from the lead‐foil roster. Resolutions from 0.6 to 5.0 lp/mm are included. Contrast is visually evaluated with a copper step‐wedge, with 17 steps of thickness 0.00 to 3.48 mm at depths of 13 mm and 5 mm.^(10)^ The readings and difference between the gray‐scale values of two specified steps is recorded for signal standardization and contrast calculation. Contrast detail inserts are visually inspected for visibility.^(11)^ Eight inserts of 10 mm diameter and depth of 0.4 to 4.0 mm are used as well as 16 inserts, one in each step‐wedge step, of 4 mm diameter at 2.5 mm depth.^(10)^ For position verification the distance between the mid‐marks on the test object and the center of the radiation limiting field is measured. The diameter of the object is also measured.^(11)^ All of these commercially available phantoms described are X‐ray imaging modality specific. In addition, several exposures are needed for comprehensive image quality control with some of these phantoms (e.g., NORMI Rad/Flu and NORMI PAS), which have loose inserts for assessing different image quality parameters. Loose inserts can be lost or damaged. The Catphan is a modular phantom and hence several different slices are needed for image quality control in CT scanning. A single modality nonspecific phantom, all‐inclusive nonmodular phantom, requiring single exposure and single slice analysis, is not currently commercially available.

Image quality is defined in terms of three parameters: contrast, spatial resolution, and noise. Image contrast is the difference in the gray scales of adjoining regions in an image. It is affected by subject, detector, and display contrast. Subject contrast is differences in signal before it is registered as part of the image. Detector contrast describes how the detector converts the signal into output and digital image. Post‐acquisition image processing affects display contrast.^(12)^ Spatial resolution describes an imaging system's capability to distinguish two closely adjoining structures as separate as they become smaller and closer together (i.e., the amount of detail in the image). It is described by a point‐spread‐function (PSF), line‐spread‐function (LSF) or edge‐spread‐function (ESF) and these are used to calculate the MTF, which shows the percentage of an object's contrast as a function of its size.^(12)^ Noise is a random “grainy” appearance in an image. Quantum noise is determined by the number of signals used to form the image and it influences the ability to detect low‐contrast objects.^(12)^ The image‐quality parameters that have to be assessed with X‐ray producing equipment in the diagnostic radiology environment include image reproducibility, circular symmetry, spatial linearity, high‐contrast resolution, low‐contrast detectability, image uniformity, spatial resolution, scaling, magnification, blurring, contrast‐detail relationships, and the presence of artifacts.^13^, ^14^ Misdiagnosis due to poor image quality is possible, causing details such as small lesions and abnormalities to be missed. Image blurring, artifacts, high levels of image noise, and poor low‐contrast detectability are examples of image‐quality degradation contributory factors.

X‐ray imaging modalities, including general X‐rays, fluoroscopy, mammography, and CT scanning, employ X‐rays for image formation. X‐rays penetrate the body and interact with tissues in order to obtain useful information about the internal anatomy, as illustrated by the produced image.^(15)^ Clinical performance of imaging systems may be assessed systemically with a good QA program. Routine image quality control (QC) compares results obtained at regular test intervals to the results obtained at acceptance testing of the equipment or to the determined baseline values. Deviations from the acceptance test or baseline values are indicative of changes in image quality. Routine QC provides a framework for continuous improvement through routine feedback and assists in identification of deviations from ideal performance (i.e., producing images with clinically relevant and diagnostically acceptable image quality). This could positively impact patient care.^(16)^

Three main problems are identified in the field of image quality assurance in resource‐limited institutions. The biggest concern is cost. The commercially available image quality assurance phantoms are expensive. Each imaging modality currently uses dedicated phantoms, hence several different phantoms are needed for comprehensive image quality assurance. Secondly, human resource deficiency, including manpower and expertise, is a limiting factor. Many institutions do not employ sufficiently trained personnel (e.g., medical physicists) to work with and analyze data from complicated commercially available phantoms. The third problem is time constraints. Image quality assurance result analyses, and decisions on needed corrective action, take time. The lack of available data analysis time is amplified by the human resource deficiency problem in resource‐limited institutions.

These limitations can be addressed by a universal phantom that would enable the required image quality assurance tests for all existing X‐ray modalities to be done. A universal image quality assurance phantom that is user‐friendly, robust, cheaper, compact, and allows for semiautomatic result analysis and recommendation of corrective action, with accompanying data analysis software, is needed. The universal image quality assurance package would include data analysis software and a user's manual explaining test objectives, phantom setup, procedures, and result analysis, as well as setting of baseline values for use in all the X‐ray imaging modalities. The aim of this research is the design, development, and manufacturing of such a phantom, of which the prototype is described here.

## II. MATERIALS AND METHODS

The image quality assurance parameters that were focused on included low‐contrast detectability, high‐contrast resolution, signal‐to‐noise ratios (SNR), contrast‐to‐noise rations (CNR), image uniformity, sensitometry for planar imaging (like general X‐rays, fluoroscopy and mammography), Hounsfield unit (HU) or CT number linearity for CT scanning and the presence of artifacts. For CT scanning, zero‐slice position and slice thickness should also be evaluated. In mammography microcalcifications, masses and fibres were assessed. The proposed phantom had to evaluate all these parameters without being a combination of the existing commercially available phantoms (i.e., it should be unique).

**Figure 1:**
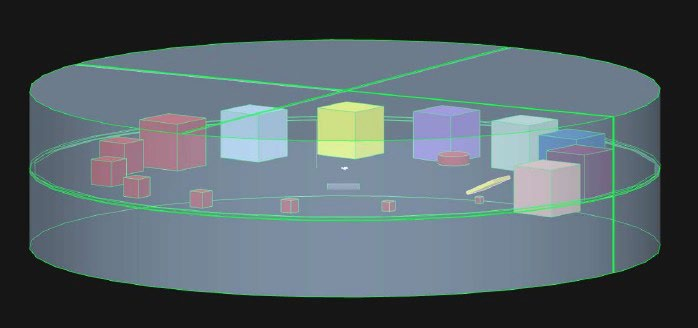
First phantom prototype 3D illustration.

Routine QC compares obtained results to set baseline or acceptance testing values and the objective is identifying deviations from these initial values. Figure 1 shows the three‐dimensional design of the first phantom prototype. The cubic inserts were arranged perpendicular to the X‐ray beam central axis in the CT scanning (upright) and planar (flat) imaging orientations, along the periphery of the phantom. Cubes made from the same material but with different sizes were used for low‐contrast detectability and mammography masses. The density of these cubes was selected to be slightly higher than that of the surrounding housing material. Cubes of the same size but made from different materials were used for HU linearity and sensitometry. Planar imaging sensitometry check assesses changes in the displayed gray scale for different materials, compared to set baseline or acceptance testing values. CT slice thickness was determined with a ramp placed in the phantom at a known angle. By using trigonometry, the slice thickness could be calculated. Mammography microcalcifications were simulated with metallic granules in different‐sized clusters and rubber bands of different diameters were used to simulate mammography fibres. A cylinder was added to assess circular geometry using on‐screen measuring tools available at X‐ray imaging units. These were also used to determine distance accuracy by measuring the distance between different inserts and comparing it to the actual known distance.

Data analysis software would employ ROI analysis to calculate SNR, CNR, image uniformity, and the MTF from the PSF of a bead or point source that was smaller than the size of a pixel. The software would require the user to input the obtained image, which is extracted from the imaging system using a step‐by‐step written standard operating procedure developed by a suitably trained person (e.g., application specialist or computer assistant). Images would be visually inspected by the observer for the presence of artifacts; for example, streaks, ghost images, and blurring. Additional 4 cm attenuator plates, made from the same material as the phantom, will be supplied to simulate patient attenuation and evaluate automatic exposure control (AEC). The first phantom prototype would therefore measure all the image quality assessment parameters, as recommended in literature.

**Figure 2:**
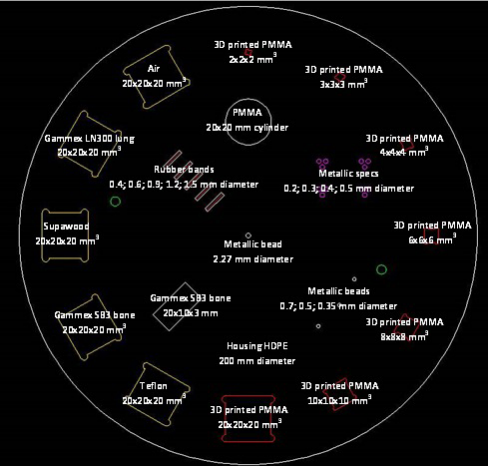
Inserts of the first phantom prototype.

The development of the initial phantom concept into the first prototype resulted in the construction of the prototype. The sizes and materials of the different phantom inserts selected for the first phantom prototype are shown in Fig. 2.

The phantom housing was made from high‐density polyethylene (HDPE) with a diameter of 200 mm and a density of 0.95 g/cm^3^. This material was selected as it was affordable, easy to machine, and had a density suitably different from that of the low‐contrast detectability cubes. The chosen diameter was sufficient to fit the inserts without interference. As the X‐ray tube circularly rotates around the phantom in CT scanning, it was designed round. The cubes for low‐contrast detectability and mammography masses were 3D printed from PMMA (density of 1.18 g/cm^3^), in sizes of 2, 3, 4, 6, 8, 10, and 20 mm^3^. The density of PMMA was 24.2 % higher than that of the HDPE housing material. HU linearity and sensitometry cubes included the 20 mm^3^ PMMA cube. Additional 20 mm^3^ cubes of Teflon (density of 2.20 g/cm^3^), Gammex SB3 bone‐equivalent plastic (density of 1.82 g/cm^3^), Supawood (density of 0.74 g/cm^3^) (Supawood Architectural Lining Systems, Robin Hill, Australia), Gammex LN300 lung‐equivalent plastic (density of 0.30 g/cm^3^) and air were incorporated. This gave six inserts ranging in density from air to bone for sensitometry assessment. Mammography fibres were simulated with 20 mm rubber bands, of density 1.26 g/cm^3^, in diameters of 0.4, 0.6, 0.9, 1.2, and 1.5 mm. The CT slice thickness ramp was a $20\times 10\times 2\;{\text{mm}}^{3}$ slab of Gammex SB3 bone‐equivalent plastic, placed in the phantom at 37°. The mammography microcalcifications were simulated with granules cut from metallic wires of diameter 0.2, 0.3, 0.4, and 0.5 mm. For MTF calculation from PSF, three metallic balls of diameters 0.35, 0.50, and 1.00 mm were included. The calculation would be done with the planned data analysis software. A 2.27 mm diameter ball bearing was included as central bead for cross‐wire centering and zero‐slice position evaluation. This ball was located exactly at the phantom center in 3D. Circular geometry was determined from a Perspex cylinder of 20 mm diameter, 20 mm length.

**Figure 3:**
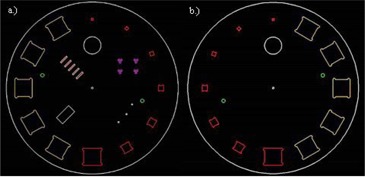
Construction drawings of first prototype: (a) bottom half, (b) top half.

Figure 3 shows the construction drawings of the first phantom prototype, as drawn in SolidWorks 2015 Premium CAD 3‐D design software (SOLIDWORKS Corp., Waltham, MA). Each of the halves was 250 mm thick. The cubic inserts, central bead, and cylinder were placed exactly on the middle plane between the two halves. The CT slice thickness ramp, mammography microcalcifications, and fibers and MTF beads were sunken into the bottom half of the first phantom prototype. In Fig. 2, semicircular cutouts are seen at the corners of the cubic inserts, due to round cutters being used to machine square voids. In the first prototype these cutouts were kept as air‐filled voids, but it could be filled with wax, with a density of 0.93 g/cm^3^, if necessary, due to image artifacts.

**Figure 4:**
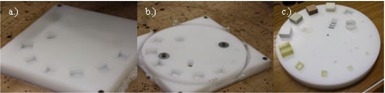
The construction process: (a) machining voids from a 250 mm slab of HDPE, (b) cutting the round first prototype phantom housing from the slab of HDPE, (c) placing the inserts in the bottom half of the first prototype phantom housing.

**Figure 5:**
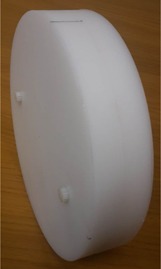
Completed first phantom prototype.

Voids were machined from a slab of phantom HDPE housing material, using a MultiCam 3000 series router (MultiCam Inc., Dallas, TX) for CNC routing. Cutting was done layer‐by‐layer and at a slow enough speed to prevent breaking of the cutters. However, cutting could not be done too slowly or else the HDPE would melt. This was done for the bottom and top halves of the phantom. Once all voids were in place, the circular phantom housing was cut from the HDPE slabs. The inserts were then fitted and the two halves were secured together using nylon screws. The process is illustrated in Fig. 4. Figure 5 shows the finished first prototype.

## III. RESULTS AND DISCUSSION

**Figure 6:**
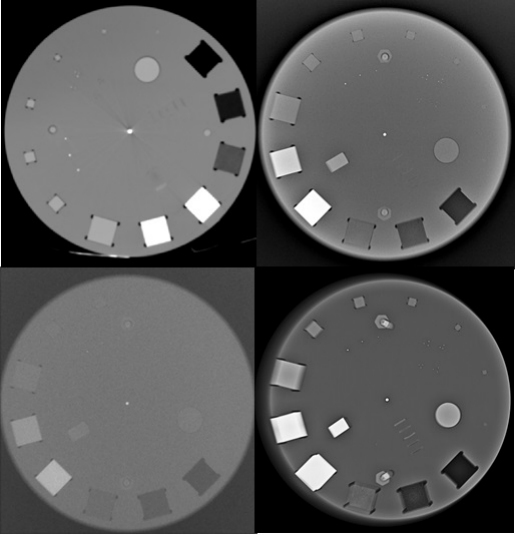
First phantom prototype: (a) CT scan axial slice, (b) general X‐ray, (c) image produced with fluoroscopy of the prototype, (d) mammogram of the prototype.

The prototype was imaged for initial testing. Figure 6 shows the original images acquired with the first prototype. Figure 6(a) is an axial CT scan slice obtained with a Siemens Somatom Definition Edge CT (Siemens Healthcare GmbH, Erlagen, Germany) using a brain‐imaging protocol; that is to say, 120 kV, 298 mA, 3.66 s, 235 cm field of view, pitch of 0.8, and 5 mm slices, and reconstruction with a medium smooth filter. A Siemens Ysio unit, at 40 kV, 2 mAs, 100 cm source‐to‐image distance, large focus, and with the cassette outside bucky, was used to produce the general X‐ray image of the first phantom prototype, illustrated in Fig. 6(b). A Siemens Axiom Luminos DRF unit was used to produce the fluoroscopic image in Fig. 6(c), with technique factors of 62.3 kV, 10.2 mA, and 0.01 ms. The mammogram in Fig. 6(d) was obtained with a Siemens Mammomat Inspiration unit at 28 kV and 62.1 mAs, using automatic exposure control. Table 1 shows visual inspection results from the images in Fig. 6. All other results, including SNR, CNR, MTF, and uniformity calculations, as well as sensitometry, geometry, and slice thickness measurements, will be done with the data analysis software, which will only form part of the final phantom package.

**Table 1:** Visual image quality assurance results from Fig. 6 images

	*Visual Image Quality Assessment Parameter*		
*Imaging Modality*	*Low Contrast Detectability*	*Artifacts*	*Other*
Mammography	$2\times 2\times 2\;{\text{mm}}^{3}$ mass seen	Geometrical distortion	0.4 mm fiber seen 0.2 mm specs seen
CT scanning	$2\times 2\times 2\;{\text{mm}}^{3}$ mass seen	Streak artifact from central ball	Not applicable
General X‐rays	$2\times 2\times 2\;{\text{mm}}^{3}$ mass seen	None	Not applicable
Fluoroscopy	8×8x8 mm^3^ mass seen	None	Not applicable

From the images in Fig. 6, the following recommendations and future improvements for subsequent phantoms were derived. The semicircular air voids at the corners of the cubic inserts did not produce significant artifacts. The nylon screws should be placed closer to the smaller cubic inserts, as these produced some interference in phantom halves fitting together. Machining should be done on a void‐by‐void basis, machining the voids marginally smaller than the actual inserts or size‐to‐size to ensure a tight fit. A 1 mm^3^ 3D‐printed PMMA cube should be added to the low‐contrast detectability and mammography mass simulation inserts.

Once these adjustments are made in the second phantom prototype, the prototype will then undergo vigorous testing, comparing its results to those from commercially available phantoms, to finalize the phantom design. The final phantom will be scientifically validated for each of the modalities to which it is intended to be applied. The validation process will include careful comparison with existing modality specific phantoms. Table 2 compares the image quality parameters that can be assessed with the universal image quality assurance phantom prototype, compared to the capabilities of the discussed commercially available phantoms. From this table it is clear that the universal image quality assurance phantom prototype is indeed an all‐in‐one image quality assurance phantom for general X‐rays, fluoroscopy, mammography, and CT scanning.

**Table 2:** Summary of commercially available modality specific phantoms compared to the universal image quality assurance phantom prototype. X indicates the parameter the phantom can assess

*Image Quality Parameter*	*Sensitometry*	*Low Contrast Detectability*	*Uniformity*	*Resolution*
ACR Mammo		X		
NORMI PAS	X	X		X
Gammex ACR CT	X	X	X	X
Catphan	X	X	X	X
CIRS 903 X‐ray		X		X
NORMI 13		X	X	X
CDRH Fluoro		X		X
NORMI Rad/Flu	X	X		X
Universal phantom	X	X	X	X
*Image Quality Parameter*	*Noise*	*Positioning and Alignment*	*Geometry and Measurement Tools*	*Artifacts*
ACR Mammo	X			X
NORMI PAS	X	X		X
Gammex ACR CT		X	X	
Catphan	X	X	X	
CIRS 903 X‐ray			X	
NORMI 13			X	X
CDRH Fluoro				
NORMI Rad/Flu		X	X	
Universal phantom	X	X	X	X
*Image Quality Parameter*	*Field Size*	*Standard Signal*	*High Contrast Resolution*	*Other*
ACR Mammo		X	X	X (Fibers)
NORMI PAS	X	X	X	X (Fibers)
Gammex ACR CT				X (Slice thickness)
Catphan				X (Slice thickness)
CIRS 903 X‐ray				
NORMI 13	X	X	X	
CDRH Fluoro				
NORMI Rad/Flu		X	X	
Universal phantom	X	X	X	X (Fibers and slice thickness)

## IV. CONCLUSION

Diagnostic radiology X‐ray images should be clinically acceptable for disease follow‐up and diagnosis. Routine image quality assurance, with a suitable phantom, ensures this. A universal phantom suitable to do quality assurance on the complete spectrum of X‐ray imaging modalities was designed and a prototype manufactured from the design. The initial images acquired with the prototype were satisfactory and showed that only small adjustments were needed to develop the prototype into a user‐friendly universal phantom. The phantom prototype described above could be seen as the leading step towards developing a universal phantom, which will fill a gap in the existing market, with special emphasis on resource‐limited institutions.

## ACKNOWLEDGMENTS

The authors acknowledge Johan Braasch, of Gebrateq Advanced Engineering, who contributed substantially to the design and development of the first phantom prototype, and wish to thank Winelands Radiology in Vergelegen Medi Clinic for imaging of the first phantom prototype. This research is presented in partial fulfilment of the requirements for the degree of PhD (Medical Physics) in the Faculty of Medicine and Health Sciences at Stellenbosch University. The research proposal is the intellectual property of Stellenbosch University under the PCT International Patent Application No. PCT/IB2016/051165.

## COPYRIGHT

This work is licensed under a Creative Commons Attribution 3.0 Unported License.

## Supporting information

Supplementary Material

Supplementary Material
