# Supplementary figures and images for: Development of a universal medical X‐ray imaging phantom prototype
Source: J Appl Clin Med Phys. 2016 Nov 8;17(6):356–65. doi: 10.1120/jacmp.v17i6.6356 (PMC5690532; doi:10.1120/jacmp.v17i6.6356)

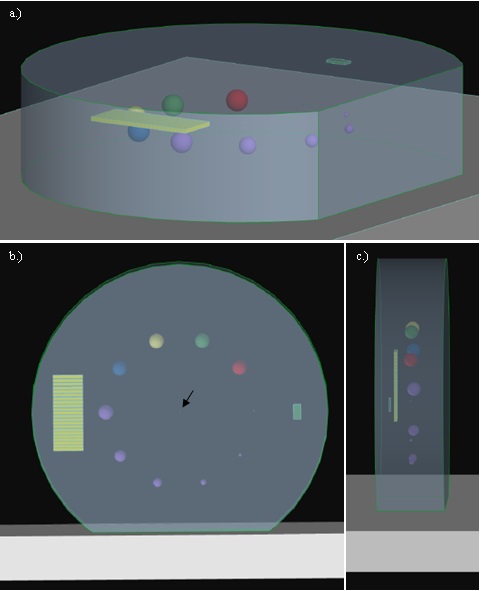

Supplement: Supplementary file 1 — Supplementary Material [file ACM2-17-356-s001.jpg]

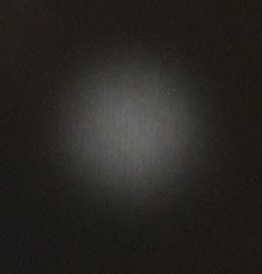

Supplement: Supplementary file 2 — Supplementary Material [file ACM2-17-356-s002.jpg]
